# A mutation in the coronavirus nsp13-helicase impairs enzymatic activity and confers partial remdesivir resistance

**DOI:** 10.1128/mbio.01060-23

**Published:** 2023-06-20

**Authors:** Samantha L. Grimes, Young J. Choi, Anoosha Banerjee, Gabriel Small, Jordan Anderson-Daniels, Jennifer Gribble, Andrea J. Pruijssers, Maria L. Agostini, Alexandra Abu-Shmais, Xiaotao Lu, Seth A. Darst, Elizabeth Campbell, Mark R. Denison

**Affiliations:** 1 Department of Pathology, Microbiology and Immunology, Vanderbilt University Medical Center, Nashville, Tennessee, USA; 2 Laboratory of Molecular Biophysics, The Rockefeller University, New York, New York, USA; 3 Tri-Institutional Program in Chemical Biology, The Rockefeller University, New York, New York, USA; 4 Department of Pediatrics, Vanderbilt University Medical Center, Nashville, Tennessee, USA; 5 Vanderbilt Institute for Infection, Immunology and Inflammation, Nashville, Tennessee, USA; Virginia Polytechnic Institute and State University, Blacksburg, Virginia, USA

**Keywords:** coronavirus, murine hepatitis virus (MHV), SARS-CoV-2, nsp13, helicase, remdesivir, antiviral resistance, nucleoside analog inhibitors, viral fitness, replicase, molnupiravir

## Abstract

Coronaviruses (CoVs) encode nonstructural proteins 1–16 (nsps 1–16) which form replicase complexes that mediate viral RNA synthesis. Remdesivir (RDV) is an adenosine nucleoside analog antiviral that inhibits CoV RNA synthesis. RDV resistance mutations have been reported only in the nonstructural protein 12 RNA-dependent RNA polymerase (nsp12-RdRp). We here show that a substitution mutation in the nsp13-helicase (nsp13-HEL A335V) of the betacoronavirus murine hepatitis virus (MHV) that was selected during passage with the RDV parent compound confers partial RDV resistance independently and additively when expressed with co-selected RDV resistance mutations in the nsp12-RdRp. The MHV A335V substitution did not enhance replication or competitive fitness compared to WT MHV and remained sensitive to the active form of the cytidine nucleoside analog antiviral molnupiravir (MOV). Biochemical analysis of the SARS-CoV-2 helicase encoding the homologous substitution (A336V) demonstrates that the mutant protein retained the ability to associate with the core replication proteins nsps 7, 8, and 12 but had impaired helicase unwinding and ATPase activity. Together, these data identify a novel determinant of nsp13-HEL enzymatic activity, define a new genetic pathway for RDV resistance, and demonstrate the importance of surveillance for and testing of helicase mutations that arise in SARS-CoV-2 genomes.

**IMPORTANCE:**

Despite the development of effective vaccines against COVID-19, the continued circulation and emergence of new variants support the need for antivirals such as RDV. Understanding pathways of antiviral resistance is essential for surveillance of emerging variants, development of combination therapies, and for identifying potential new targets for viral inhibition. We here show a novel RDV resistance mutation in the CoV helicase also impairs helicase functions, supporting the importance of studying the individual and cooperative functions of the replicase nonstructural proteins 7–16 during CoV RNA synthesis. The homologous nsp13-HEL mutation (A336V) has been reported in the GISAID database of SARS-CoV-2 genomes, highlighting the importance of surveillance of and genetic testing for nucleoside analog resistance in the helicase.

## INTRODUCTION

Coronaviruses (CoVs) are a family of single-stranded, positive-sense, enveloped RNA viruses that belong to the viral order *Nidovirales*. CoVs infect a wide range of avian and mammalian hosts and are divided into four genera: alphacoronaviruses, betacoronaviruses, gammacoronaviruses, and deltacoronaviruses. Members of the alphacoronavirus and betacoronavirus genera cause disease in humans that range from mild to severe upper respiratory distress (1 - 6). Betacoronaviruses include the highly pathogenic severe acute-respiratory syndrome-related coronavirus (SARS-CoV), the Middle East respiratory severe acute-respiratory syndrome-related virus (MERS-CoV), and the causative agent of the COVID-19 pandemic, SARS-CoV-2 (4 - 6). This genus also includes the model nonhuman CoV murine hepatitis virus (MHV). CoVs have caused three outbreaks of severe human diseases in the last two decades, and though multiple vaccines and therapeutics are available, COVID-19 remains a global public health threat. Further, there remains the possibility that future emergent novel CoVs may cause severe human disease.

There are three antivirals in use for the treatment of people hospitalized with COVID-19: the protease inhibitor Paxlovid, and the nucleoside analogues molnupiravir (MOV) and remdesivir (RDV) (7 - 9). RDV is the first Food and Drug Association (FDA)-approved antiviral used to treat patients hospitalized with SARS-CoV-2 infection (10). RDV is an adenosine nucleoside analogue with broad-spectrum activity against several RNA viruses, including human and zoonotic CoVs (9, 11 - 18).

Therapeutic administration of RDV has been shown to reduce viral load and improve disease outcomes in animal models and has been used to treat COVID-19 in humans since its Emergency Use Authorization by the FDA on 1 May 2020 and full approval in October 2020 (9, 19 - 23). RDV resistance mutations have been reported in MHV, SARS-CoV, MERS-CoV, and SARS-CoV-2, including at low levels in more than 15 million SARS-CoV-2 genomes reported in the GISAID database (24). To date, all reported CoV RDV-resistance mutations have been identified in the nonstructural protein 12 RNA-dependent RNA polymerase (nsp12-RdRp), supporting multiple potential mechanisms for the RDV antiviral mechanism of action (14, 24 - 30).

The triphosphate form of RDV (RDV-TP) inhibits RNA synthesis by acting as a competitive substrate for adenosine incorporation into nascent RNA. In SARS-CoV-2, nsp12-RdRp selectively incorporates RDV-TP over ATP (18, 31). One mechanism of action for CoV replication inhibition by RDV is that the nsp12-RdRp incorporates RDV-monophosphate (RMP) into the product RNA, followed by three subsequent nucleotides before stalling or pausing on the template strand, and leading to premature termination of synthesis (32, 33). Polymerase pausing and premature synthesis are evident only at low concentrations of NTPs (~10 μM) and can be overcome by higher NTP concentrations (25). The second reported RDV mechanism of action is second-strand inhibition, in which RDV-MP is incorporated into product RNA that acts as a template for future synthesis by the RdRp, where template-dependent inhibition of the nsp12-RdRp can occur (25). Mutations in the nsp12-RdRp have been identified that reduce the concentration of uridine triphosphate required to overcome template-dependent inhibition (24). The impact of RDV on other CoV replication proteins and alternate pathways for RDV resistance in other nsps have not been reported and are unknown.

**Fig 1:**
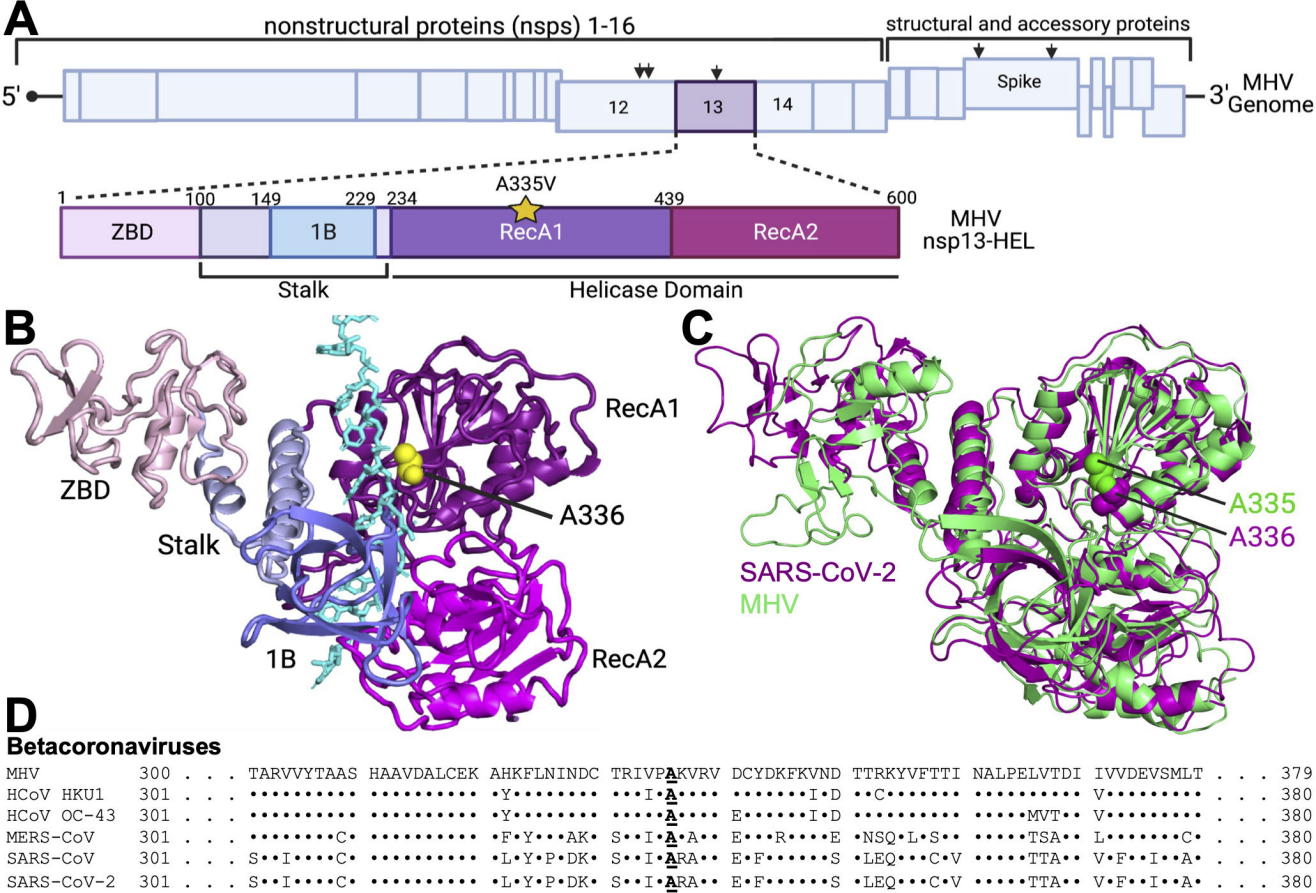
MHV helicase substitution A335V occurs at a conserved, surface-exposed residue of the CoV helicase RecA1 domain. (**A**) Schematic of the MHV genome with sites of nonsynonymous mutations selected during MHV passage with GS-441524 (black arrows). Below, a schematic of the MHV nsp13-HEL showing the helicase domains: (i) the Nidovirus-specific zinc-binding domain (ZBD; pink), (ii) the stalk (light purple), (iii) 1B beta barrel (blue), (iv) RecA1 (purple), and (v) RecA2 (magenta). A335V is in the RecA1 domain (yellow star). Figure made with Biorender. (**B**) The previously described cryoEM structure of the SARS-CoV-2 nsp13-HEL (PDB: 6XEZ) (38) in complex with nsp7/8_2_/12 (not shown) and RNA (cyan) was used to map the location of the A336 residue (yellow spheres). Protein domains are colored to match the MHV nsp13-HEL domains shown in the schematic in (**A**). (**C**) Alignment between the PyMOL model of the MHV nsp13, generated using ColabFold v1.5.2 (39)(green) and the SARS-CoV-2 structure used in (**C**) (purple). The RMSD value of the alignment is 3.364. (**D**) Amino acid conservation of A335 and surrounding region across divergent betacoronaviruses. Sequence identity to MHV is shown by dots.

CoVs encode single-stranded RNA genomes that range in length from 26 kb to 32 kb (34). The 5′ two-thirds of the genome encodes nonstructural protein domains 1–16 (nsps1-16), while the 3′ third of the genome encodes structural and accessory proteins (35, 36) (Fig. 1A). CoVs are unique in that they perform both continuous and discontinuous RNA synthesis; CoVs use continuous synthesis to produce new genomic RNAs (gRNA) and discontinuous synthesis to produce nsps and structural and accessory proteins in a set of nested subgenomic RNAs that contain identical 5′ leader sequences and common 3′ ends (37).

CoV replication is initiated in cells by translation of the infecting genome (+)RNA, which is translated from two open reading frames (ORFs), ORF1a and ORF1ab (37). Translation of ORF1a yields a ~400 kDa polyprotein 1 a (pp1a), and a ribosomal frameshift at the 3′ end of ORF1a results in a less abundant ~700 kDa pp1ab (37). The pp1a comprises the nsp1-11 domains, while pp1ab contains the nsp1-10 and 12–16 domains (34). Each nsp domain is flanked by conserved protease cleavage sites that are cleaved by protease activities in nsp3 (PLpro) and nsp5 (3CLpro/Mpro) to release the individual nsps (34, 37). The nsps 7–16 are involved in CoV RNA synthesis and processing, and structural and biochemical data suggest that a larger complex forms between the RNA and the nsp12 RNA-dependent RNA polymerase (nsp12-RdRp), nsps 7 and 8 (cofactors for nsp12), and the nsp13 helicase (nsp13-HEL) (40). This multi-protein replication-transcription complex (RTC) is also thought to associate with other nsps, including the proofreading nsp14 5′-3′ exoribonuclease and N7-methyltransferase (nsp14-ExoN) (41). In CoVs, a complex of nsps 7 and 8 and the nsp12-RdRp can bind RNA and has been shown to be sufficient for *in vitro, de novo* initiation of RNA synthesis in the presence of Mg^2+^ (40 - 42). A complex of nsps 7, 8, and 12 can associate with the nsp13-HEL, and this association has been shown to improve helicase unwinding activity (43, 44).

The CoV nsp13-HEL, a superfamily 1b (SF1b) helicase, unwinds both dsRNA and dsDNA with a 5′-3′ polarity using diverse NTPs as substrate (38, 45 - 49). In the CoV helicase, the unwinding and NTPase activities are uncoupled; NTPase activity can occur in the absence of nucleic acid unwinding (43). In addition to its unwinding, translocation, and NTPase activity, multiple additional functions have been attributed to the CoV helicase, including 5′-triphosphatase activity, which likely plays a role in RNA capping, interferon antagonism, and a role in tissue tropism (48, 50, 51). The CoV nsp13-HEL comprises five domains: a zinc-binding domain (ZBD; unique to nidoviruses) and two core catalytic domains, RecA1 and RecA2, which are connected to the ZBD by the stalk domain and 1B domain (38, 45, 46) (Fig. 1A and B). Cryo-EM structures of the SARS-CoV-2 nsp13-HEL in complex with nsp7 and two copies nsp8 and the nsp12-RdRp (nsps 7/8_2_/12) provide structural evidence for coupling between the nsp12-RdRp and nsp13-HEL and between nsp8 and nsp13-HEL (42, 43, 52 - 54). These studies, along with previous structures of the SARS-CoV and MERS-CoV nsp13-HELs, support previous work identifying a functional relationship between the nsp12-RdRp and the nsp13-HEL (42, 44). However, the impact of RDV on other proteins in the RTC and alternative pathways for RDV resistance in other nsps has not been reported.

Prior to the emergence of SARS-CoV-2, we passaged the model CoV MHV in the presence of GS-441524, the parent compound of RDV, to define genetic pathways and specific mutations mediating RDV resistance (14). After establishing phenotypic resistance in the passaged population, we sequenced the viral populations and identified three nonsynonymous mutations in the replicase proteins: two mutations in the nsp12-RdRp (F476L and V553L) and one mutation in the nsp13-HEL (A335V), as well as two mutations in the Spike glycoprotein (A34V and I924T) (Fig. 1A) (14). Our initial focus was on the nsp12-RdRp V553L and F476L substitutions, which we showed conferred partial RDV resistance alone and when combined in an isogenic cloned MHV (14). The contribution of the nsp13-HEL substitution, A335V, to RDV resistance was not further determined in that study.

Here, we show that the MHV nsp13-HEL A335V substitution mediated partial RDV resistance independent of the previously characterized nsp12-RdRp mutations F476L and V553L. Virus with the A335V substitution had replication kinetics similar to WT-MHV but had no fitness advantage in direct competition with WT-MHV. Further, MHV with the A335V substitution remained sensitive to the cytidine nucleoside analog antiviral MOV. Biochemical analyses of the SARS-CoV-2 nsp13-HEL encoding the homologous A336V substitution showed that the mutant helicase remained able to associate with an nsp7/8_2_/12 complex, but that the unwinding rates for dsDNA and dsRNA were reduced compared to the WT nsp13-HEL. The SARS-CoV-2 nsp13 with the A336V substitution also had decreased ATPase activity compared to WT SARS-CoV-2 nsp13-HEL. These results support important roles for the CoV helicase in viral RNA synthesis and now specifically in RDV resistance.

## RESULTS

### MHV helicase substitution A335V occurs at a conserved, surface-exposed residue of the CoV helicase RecA1 domain

Passage ofMHV in the nucleoside analog GS-441524, the parent compound of remdesivir (RDV) selected for a mutation in the nsp13-helicase (nsp13-HEL), resulted in an A335V substitution as previously reported (14). We used sequence alignment to determine that A335 is located in the helicase RecA1 domain but not in a known helicase catalytic motif (Fig. 1A). No structure of the MHV nsp13-HEL has been reported, so we used available crystal and cryoEM structures of SARS-CoV, MERS-CoV, and SARS-CoV-2 to predict the location of the substituted residue on the CoV helicase structure (43, 52 - 56). We determined that A335 (A336 in SARS-CoV-2) is in a surface-exposed loop of RecA1 that is adjacent to the RNA-binding channel (Fig. 1B). We then performed *de novo* modeling of the MHV nsp13-HEL using ColabFold, a software for modeling protein folding predictions, and aligned this model with the published SARS-CoV-2 cryoEM structure (Fig. 1C) (57, 58). While the amino acid identity between MHV and SARS-CoV-2 is ~68%, the structure alignment shows a highly conserved overall structure with a Root Square Mean Deviation (RMSD) of 3.364, where most of the variation occurs in the flexible ZBD (Fig. 1C and D). The A335 residue is conserved at the homologous location across divergent betacoronaviruses, and the surrounding amino acid sequence has high sequence similarity (Fig. 1D).

### The order of emergence and coincidence of the MHV nsp13-HEL A335V substitution and the nsp12-RdRp substitutions

**Fig 2:**
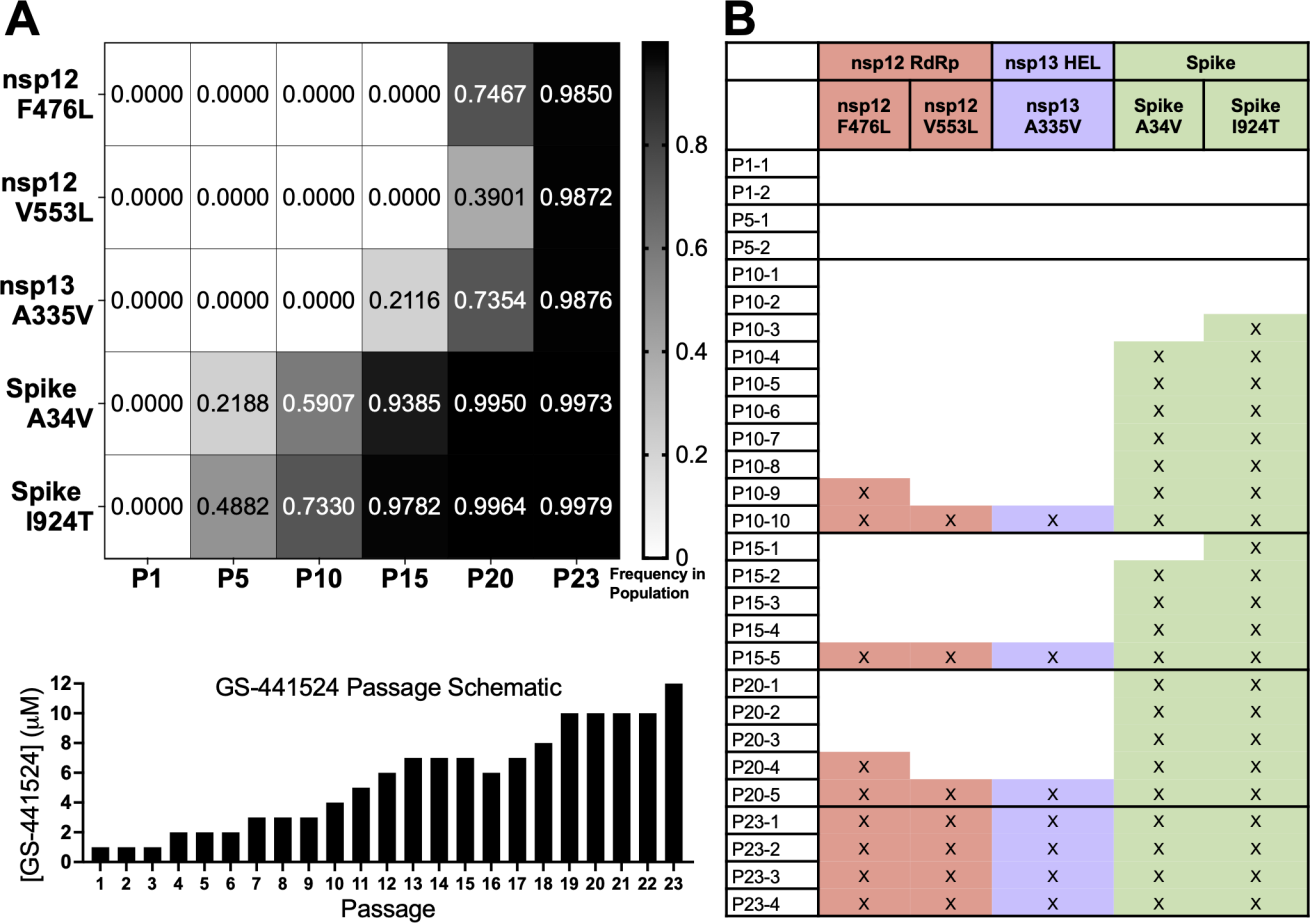
The order of emergence and coincidence of the MHV nsp13-HEL A335V substitution and the nsp12-RdRp substitutions (**A**) RNA sequencing of DBT-9 cell monolayers infected with the indicated passage virus at an MOI of 0.01 PFU/cell. Samples were aligned to the MHV genome (AY910861), *n* = 1. Below, schematic showing the concentration of GS-441524 used during passage. Concentration of GS-441524 was increased when the cytopathic effect remained at least 80%. (**B**) Table of Sanger Sequencing results from virus isolated from triple plaque purification.

To better understand the contributions of the nsp12-RdRp and nsp13-HEL mutations to RDV resistance, we determined the order in which they emerged during passage using Illumina RNA sequencing of total RNA from virus-infected DBT cell monolayers of passages 1, 5, 10, 15, 20, and 23. The results showed that the nsp13-HEL A335V substitution but not the nsp12-RdRp mutations was detected by passage 15. The nsp12-RdRp substitutions (F476L and V553L) were observed at passage 20 (Fig. 2A). By passage 23, the nsp12-RdRp and nsp13-HEL substitutions were present at a frequency greater than 0.98. The Spike mutations A34V and I294T were already present by passage 5 and likely reflect nonspecific tissue culture adaptation (Fig. 2A).

While total RNA sequencing depth is a sensitive indicator for the detection of even minor variants or new mutation selection, it cannot be used to understand the co-occurrence of mutations in viral genomes. We next tested the coincidence of the replicase mutations in the nsp12-RdRp and nsp13-HEL mutations during selection. Virus plaques from passages 1, 5, 10, 15, 20, and 23 were plaque purified three times and then P1 stocks from each plaque were sequenced by di-deoxy (Sanger) sequencing. The three replicase mutations nsp12-RdRp F476L and V553L substitutions and nsp13-HEL A335V substitution were identified together in one or more plaques at passages 10, 15, 20, and 23, alongside the Spike cell culture mutations (Fig. 2B). In contrast to the RNA-seq analysis of the population lineages, in plaque purified expanded virus populations, all mutations could be identified by passage 10. When the nsp13-HEL A335V mutation was identified in plaque purified viruses, it was always co-detected with both nsp12-RdRp substitutions (Fig. 2B). Thus, the nsp13-HEL A335V substitution was either co-selected or selected prior to the nsp12-RdRp mutations, suggesting selective pressure for this mutation.

### nsp13-HEL A335V confers partial RDV resistance in MHV

**Fig 3:**
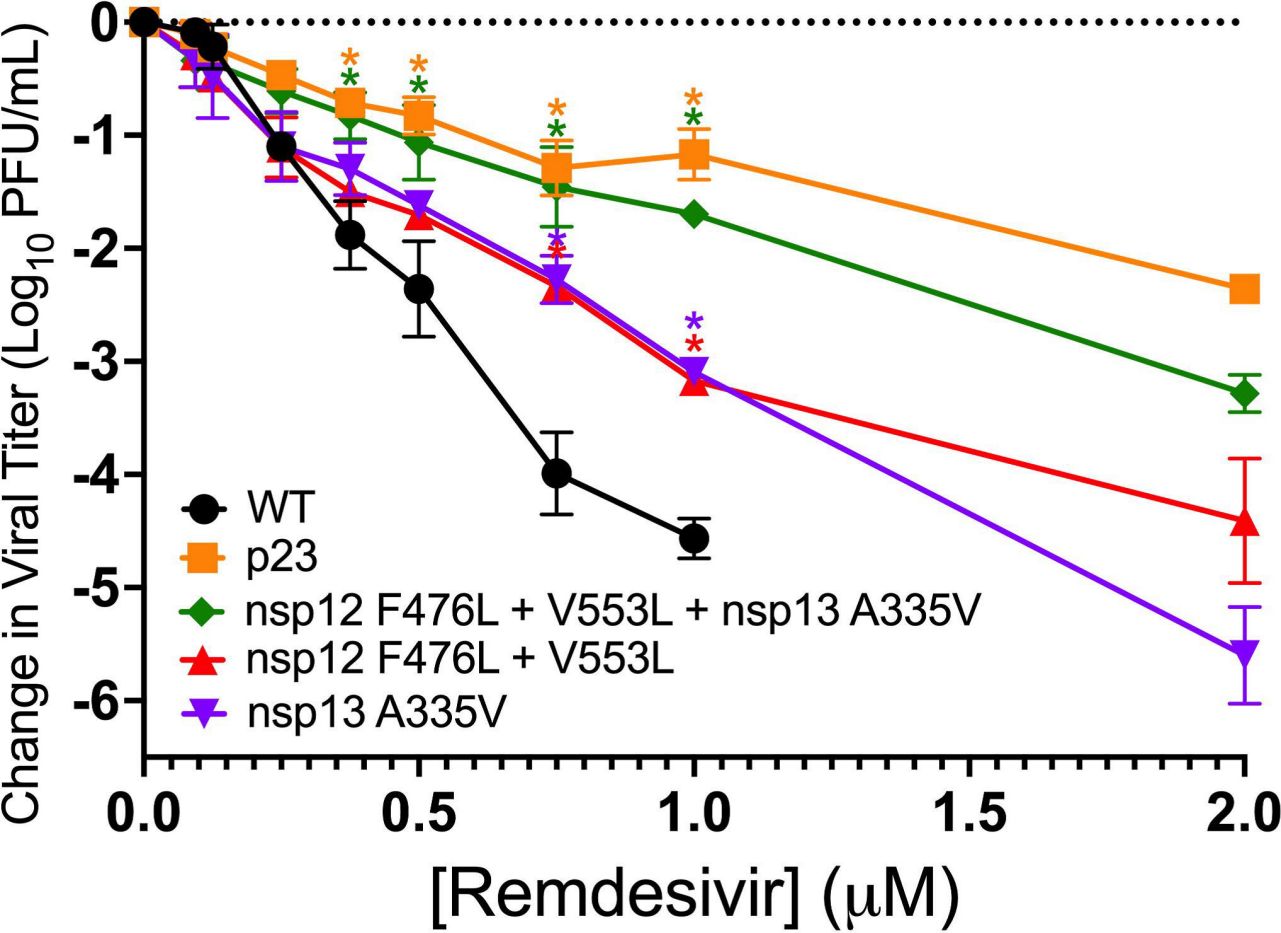
The nsp13-HEL A335V substitution confers independent partial resistance to RDV in MHV. Change in viral titer of WT, passage virus p23, nsp13-HEL A335V, nsp12-RdRp F476L/V553L, and nsp12-RdRp F476L/V553L + nsp13-HEL A335V. Virus titer was determined by plaque assay. Data are means ± SEM from three independent experiments, each with three replicates. Statistical significance compared to WT at each concentration was determined by one-way ANOVA at each concentration, with correction for multiple comparisons and is denoted by asterisks: *, *P* < 0.05.

We next tested the contribution of the nsp13-HEL A335V mutation to the RDV-resistance phenotype of the population virus, passage 23 (p23). We engineered the mutation encoding the A335V substitution into an isogenic WT-MHV background alone and in combination with the nsp12-RdRp mutations F476L and V553L. We then infected cells at a multiplicity of infection (MOI) of 0.01 PFU/cell, treating with RDV dilutions. The population virus p23 and the MHV mutants encoding the nsp13-HEL A335V, nsp12-RdRp F476L/V553L, and the triple-mutant virus (encoding nsp12-RdRp F476L/V553L + nsp13-HEL A335V) were less sensitive to RDV compared to WT (Fig. 3). The population virus from passage (p23) and the MHV encoding all three substitutions were less sensitive than WT-MHV at 0.375 µM, 0.5 µM, 0.75 µM, and 1 µM RDV, while the nsp13-HEL A335V mutant virus and the nsp12-RdRp F476L/V553L mutant virus were statistically less sensitive than WT-MHV at 0.75 µM and 1 µM RDV (Fig. 3). All mutant viruses were still detectable following infection in the presence of 2 µM RDV, while WT-MHV was not detected (Fig. 3). Thus, the nsp13-HEL A335V substitution alone was sufficient to confer partial RDV resistance and MHV with all three replicase substitutions closely recapitulated the resistance phenotype of the p23 passage population virus.

### Replication and fitness of MHV with the nsp13-HEL A335V substitution

**Fig 4:**
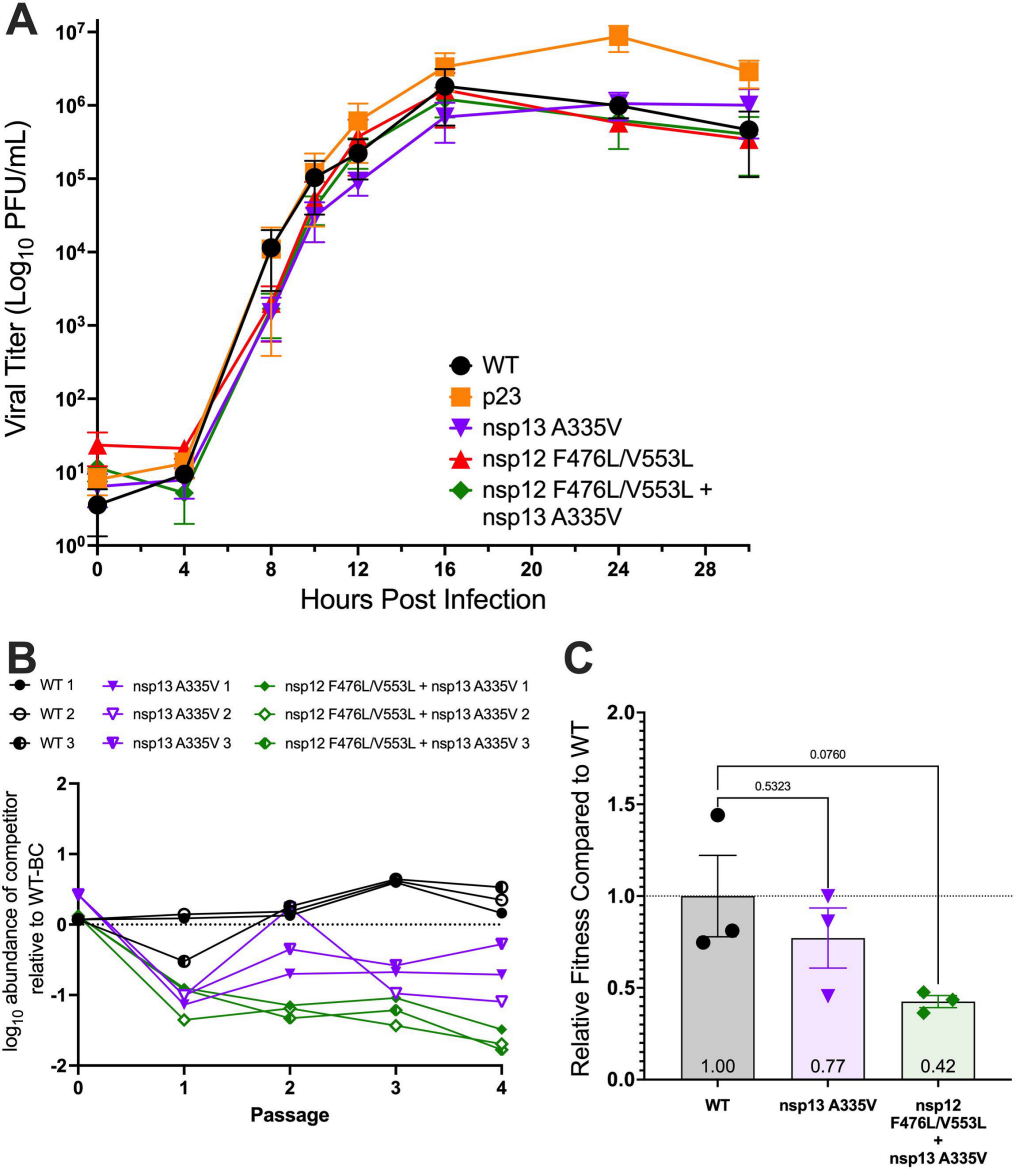
MHV with the nsp13-HEL A335V substitution is replication competent but has impaired fitness. (**A**) Multi-cycle replication kinetics of WT, passage virus p23, nsp13-HEL A335V, nsp12-RdRp F476L/V553L, and nsp12-RdRp F476L/V553L + nsp13-HEL A335V. DBT-9 cells were infected with the indicated virus at an MOI of 0.01 PFU/cell . Supernatant samples were collected at the indicated times, and the virus titer was determined by plaque assay. Data shown are from three independent experiments, each with three replicates. Points represent experiment means ± SEM. (**B**) DBT-9 cells were co-infected with a barcoded (BC) WT MHV and nonbarcoded WT, nsp13-HEL A335V, p23, or nsp12-RdRp F476L/V553L + nsp13-HEL A335V at a combined MOI of 0.1 PFU/cell. The resulting supernatants were passaged four times. The relative quantities of barcoded and nonbarcoded cDNAs were plotted over passage for the three independent lineages of each competition. (**C**). Linear regression from panel B was used to determine relative fitness for each nonbarcoded virus. Individual data (*n* = 3) are graphed (means ± SEM) and statistical significance compared to WT was tested by one-way ANOVA.

We first determined replication kinetics and peak titers for MHV with the A335V substitution alone and in combination with nsp12-RdRp F476L/V553L. The A335V mutant virus achieved similar kinetics and maximum titers as WT-MHV (Fig. 4A). Similarly, MHV encoding both nsp12-RdRp substitutions and nsp13-HEL A335V achieved titers and replication kinetics similar to WT-MHV (Fig. 4A). To assess the competitive fitness of virus containing nsp13-HEL A335V alone or in combination with the nsp12-RdRp substitutions, we coinfected WT or mutant MHV with a barcoded MHV containing seven synonymous mutations in nsp2, allowing barcoded and competitor virus to be independently quantified by RT-qPCR (58). Barcoded WT and competitor viruses were infected at a 1:1 ratio with a total MOI of 0.1 PFU/cell, and the supernatant virus from the initial infection was serially passaged four times. Throughout passage, the nsp13-HEL A335V substitution did not confer a fitness advantage compared to WT-MHV, either alone or in combination with nsp12-RdRp F476L/V553L substitutions (Fig. 4B and C).

While the mutant competitors had less-abundant genomes compared to barcode over passage and the ratios after a single passage showed a decrease in the ratio of mutant competitor RNA compared to barcoded WT (Fig. 4B), linear regression analysis did not result in significant difference in fitness relative to WT (Fig. 4C). Therefore, we conclude that A335V alone or in combination does not confer a fitness advantage compared to WT MHV.

### The nsp13-HEL A335V substitution does not confer cross-resistance to the parent compound of molnupiravir

**Fig 5:**
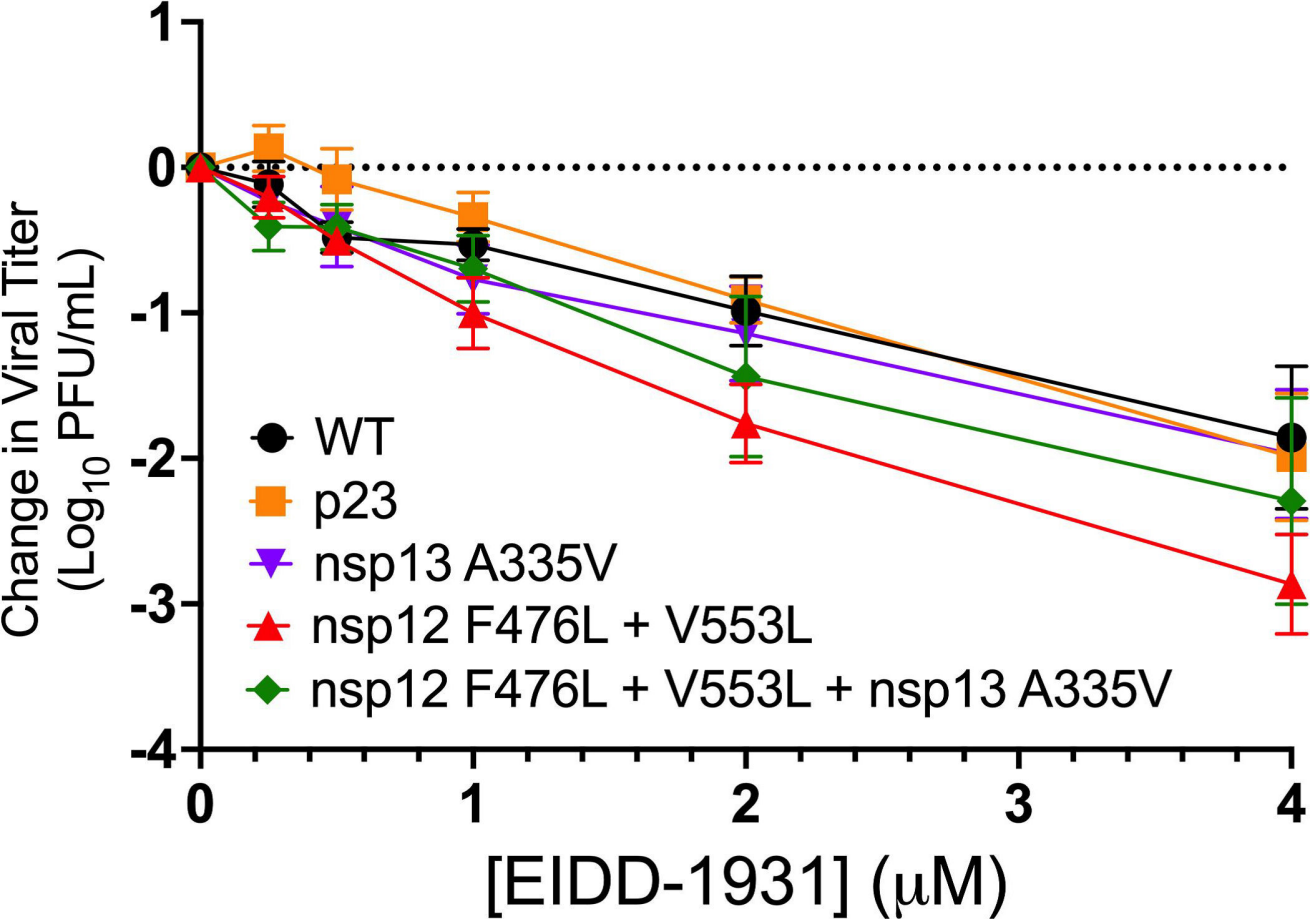
nsp13-HEL A335V does not confer cross-resistance to the nucleoside analog antiviral EIDD-1931. Change in viral titer in response to varying concentrations of the active form of the nucleoside analog molnupiravir, EIDD-1931, for WT, passage virus p23, nsp13-HEL A335V, nsp12-RdRp F476L + V553L, and nsp12-RdRp F476L + V553L + nsp13-HEL A335V. Virus titer was determined by plaque assay. Data are means standard deviations from three independent experiments, each with three replicates. Statistical significance compared to WT at each concentration was determined by one-way ANOVA, with correction for multiple comparisons. All viruses were not statistically significant from WT.

To determine if the resistance phenotype was specific to RDV, an adenosine analog, we tested virus sensitivity to EIDD-1931, the active form of the antiviral MOV. EIDD-1931 is a cytidine analog with broad-spectrum activity against CoVs that impairs viral replication by increasing mutagenesis during replication (59, 60). We infected with WT and mutant viruses and treated cells across a range of EIDD-1931 concentrations. All viruses with RDV resistance mutations demonstrated WT-like sensitivity to EIDD-1931 (Fig. 5A). These data suggest that the RDV resistance conferred by the nsp13-HEL A335V substitution was specific to RDV.

### The homologous substitution A336V is reported as a naturally occurring mutation in SARS-CoV-2

Due to the conservation of the A335 residue across betacoronaviruses and the continued use of RDV as a monotherapy, we next determined if the substitution at the homologous residue, A336, was present in reported SARS-CoV-2 genome sequences. We conducted a literature review of reported RDV resistance and searched the repository of sequences in GISAID for variants at the A336 residue. In the literature, mutations at A336 have not been reported in case studies of RDV resistance in patients or *in vitro* passaging of SARS-CoV-2 in the presence of RDV. Our search in the GISAID found 9,373 viruses with mutation at A336 from the over 15.4 million virus sequences in the database (23). Of the variants at A336, there were 888 virus sequences with an A336V substitution (23). The earliest report of a SARS-CoV-2 virus with an A336V mutation was from a collection that took place in April 2020 (23). These reports demonstrate that while A336V occurs at a very low frequency, it is a naturally occurring polymorphism in SARS-CoV-2.

### SARS-CoV-2 helicase with the A336V substitution associates with other RTC proteins

**Fig 6:**
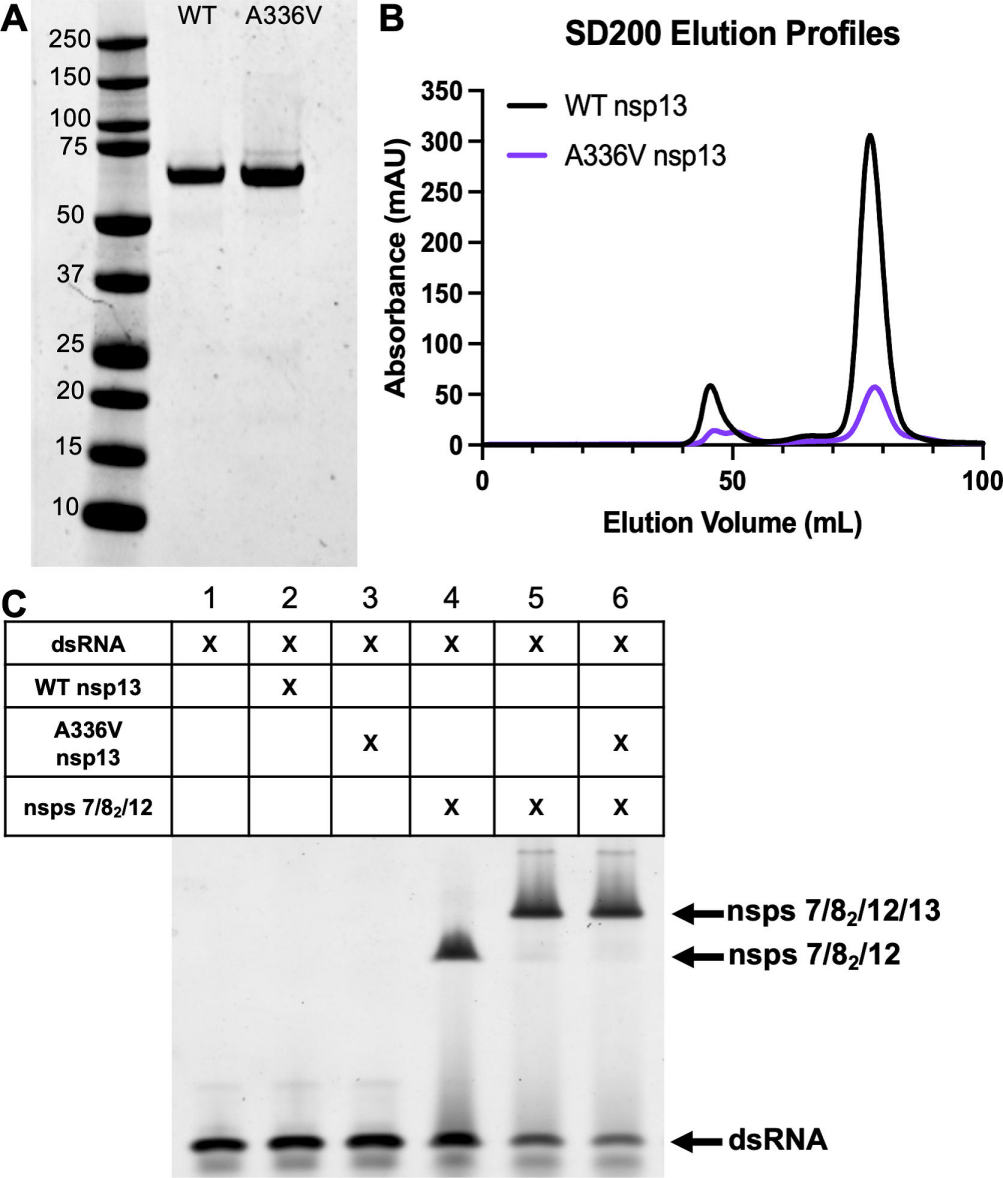
SARS-CoV-2 helicase with the A336V substitution remains capable of association with other members of the RTC. (**A**) SDS-PAGE of purified WT nsp13-HEL and A336V nsp13-HEL. (**B**) Gel filtration traces for WT and A336V nsp13-HEL. nsp13-HEL peaks observed at 67 kDa. (**C**) WT and mutant nsp13-HEL were pre-incubated with ADP-AIF3 and dsRNA alone and with purified nsps 7/8_2_/12. Native electrophoretic mobility shift assay (EMSA) visualizing the formation of the nsp 7/8_2_/12 + RNA (lane 4). When WT or mutant nsp13-HEL is added, a stable complex of nsps 7/8_2_/12/13 is upshifted similarly. Complexes were separated on a 4.5% polyacrylamide gel and stained with Gel Red to detect RNA.

While no biochemical systems for MHV nsp13-HEL and 7/8_2_/12 are available, SARS-CoV-2 replication protein biochemical systems are now well developed. Since SARS-CoV-2 nsp13-HEL A336V is a natural polymorphism, we sought to determine if this system could assess the impact of the A336V substitution on nsp13-HEL association with other members of the RTC. We tested the impact of the homologous A336V substitution in the SARS-CoV-2 helicase. The A336 residue is in the RecA1 domain, which is not predicted to be an interface site with other members of the RTC (47). To determine if A336V affects RTC formation, we expressed and purified WT nsp13-HEL and A336V nsp13-HEL as previously described (43, 53) (Fig. 6A and B). WT and mutant nsp13-HEL were pre-incubated with ADP-AIF3, followed by a native electrophoretic mobility shift assay (EMSA) on nsp13-HEL incubated with only dsRNA or in the presence of pre-formed complexes of nsp7/nsp8/nsp12-RdRp (Fig. 6C). Both WT and A336V nsp13-HEL were able to form a stable association with nsp7/8_2_/12-RdRp (Fig. 6C).

### SARS-CoV-2 helicase with the A336V substitution has a reduced unwinding rate compared to WT helicase

**Fig 7:**
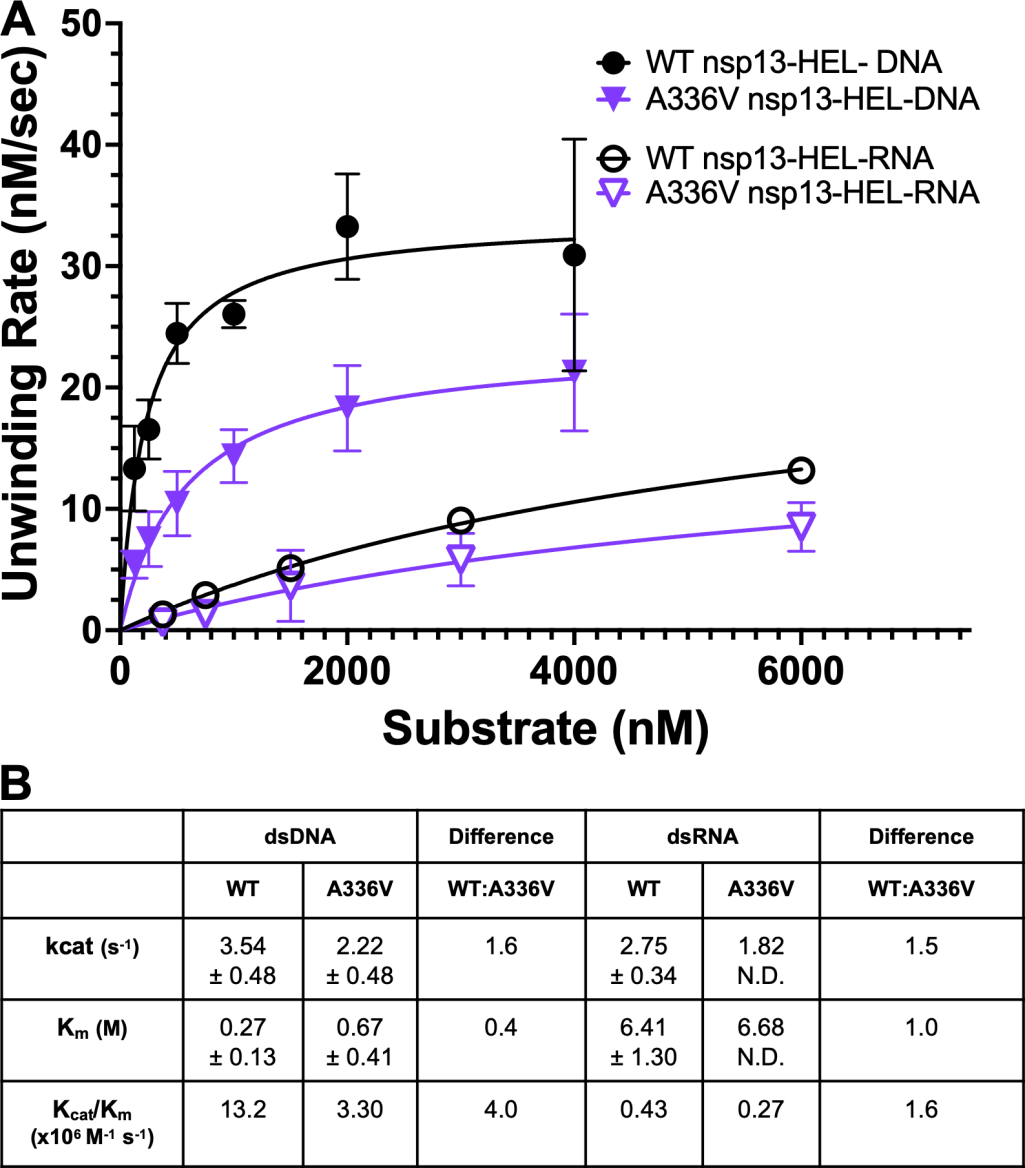
SARS-CoV-2 helicase with the A336V substitution has a reduced unwinding rate. (**A**) Michaelis-Menten curve of helicase unwinding rate with dsDNA and dsRNA substrates. Initial unwinding velocities were graphed across varying concentrations of DNA (0–4 M) for 10 nM of WT or A336V nsp13-HEL. Points and error bars indicate the average and SD, respectively. *n* = 4 (DNA) and *n* = 3 (RNA). (**B**) Table of kinetic values derived from fitting unwinding rates to Michaelis-Menten kinetics. Values shown represent the calculated 95% confidence intervals for each parameter unless denoted N.D. (not determined).

We next tested whether the A336V substitution altered the enzymatic activity of the purified nsp13-HEL *in vitro*. Based on its location in the RNA-binding channel, we hypothesized that A336V might alter RNA binding and consequently alter unwinding activity. We performed an unwinding assay with both dsRNA and dsDNA (Fig. 7A). The initial rates of dsRNA unwinding remained linear at the highest substrate concentration, preventing a quantitative analysis of the impact of the A336V substitution on RNA unwinding activity despite a trend toward impaired unwinding (Fig. 7A). With dsDNA, WT nsp13-HEL has a k_cat_/K_m_ of 13.2 ± 6.6 (M^−1^s^−1^), while A336V nsp13-HEL has a k_cat_/K_m_ of 3.3 ± 2.1 (M^−1^s^−1^), a fourfold reduction in catalytic activity (Fig. 7B). These data demonstrate that the A336V substitution impaired helicase unwinding activity *in vitro*.

### A336V reduces SARS-CoV-2 helicase ATPase activity

**Fig 8:**
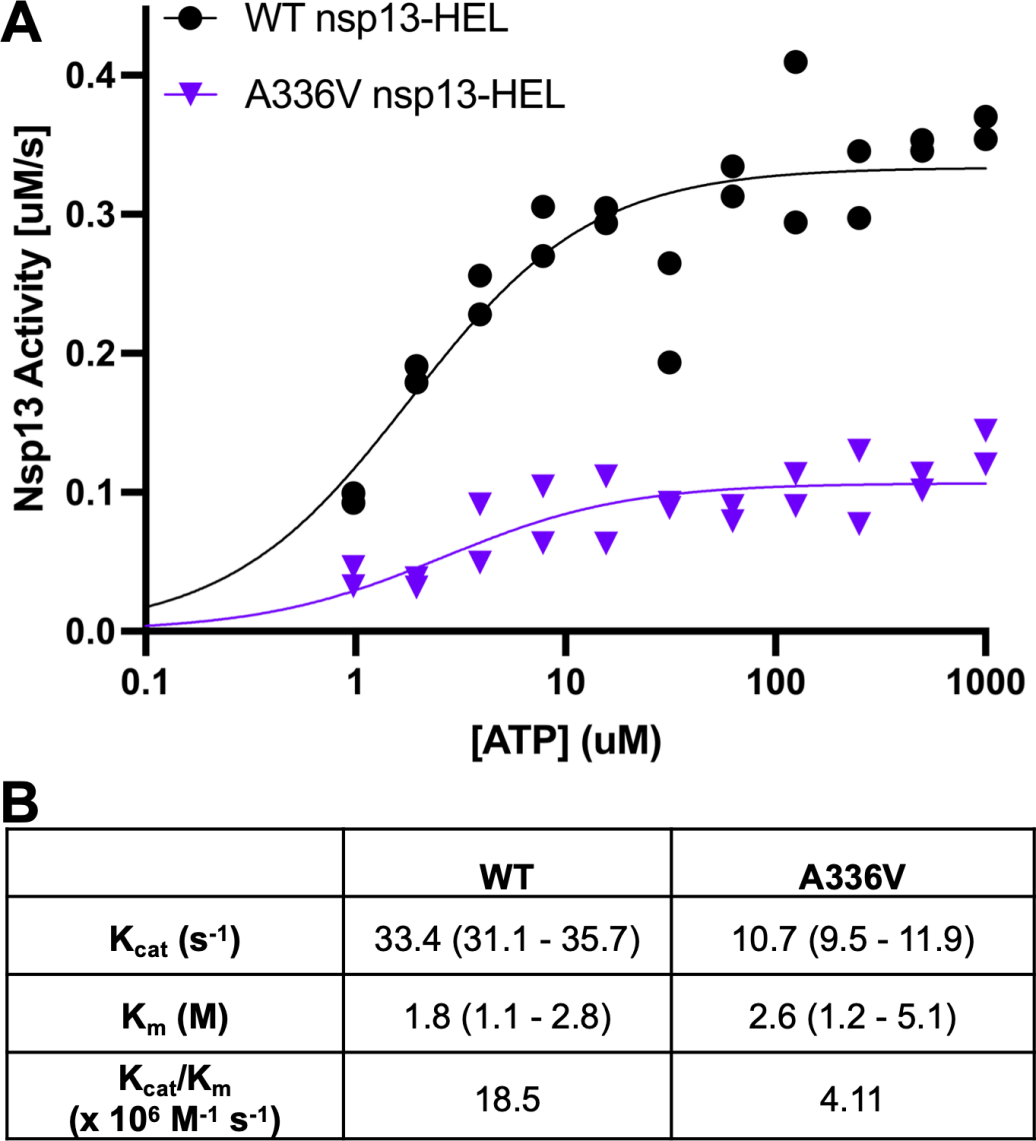
SARS-CoV-2 helicase with the A336V substitution has reduced ATPase activity. (**A**) Michaelis-Menten curve of ATPase activity of WT and A336V nsp13-HEL. Points and error bars indicate the average and SD, respectively. *n* = 2. (**B**) Table of kinetic values derived from fitting ATPase rates to Michaelis-Menten kinetics. Values shown represent the calculated 95% confidence intervals for each parameter.

CoVs are a virus family in the viral order *Nidovirales*. In CoVs and the related *arteriviridae* family viruses, helicase NTPase activity is uncoupled from unwinding activity, allowing an assessment of these enzymatic activities independently (43, 61 - 63). Solved structures of the SARS-CoV-2 helicase show that A336 is located over 27 Å away from the nucleotide binding pocket, so we did not expect the alanine to valine substitution to impact ATPase activity (43, 53). ATP consumption by the SARS-CoV-2 helicase in the absence of dsRNA or dsDNA was assessed using an NADH-coupled ATPase assay (49). Similar to unwinding activity, the mutant nsp13-HEL had a ~4.5-fold reduced ATPase activity (k_cat_/k_m_), an effect that was largely mediated through a change in k_cat_ and not k_m_ (Fig. 8B).

## DISCUSSION

As SARS-CoV-2 continues to evolve variants that elude natural immunity, vaccine-derived immunity, and available monoclonal antibodies, the development and use of antivirals targeting conserved viral replication function remains critical. RDV remains important in this arsenal of direct-acting antivirals. The pathways to RDV resistance are being investigated *in vitro* and by studying genomes isolated from patients treated with RDV (14). In CoVs, mutations in the nsp12 RNA-dependent RNA polymerase (nsp12-RdRp) have been shown to be responsible for resistance to nucleoside analogs (14, 24 - 29). RDV resistance mutations have been reported in the nsp12-RdRp in MHV, SARS-CoV, MERS-CoV, and SARS-CoV-2 (14, 24 - 29). Demonstrated mechanisms of RDV resistance include altering the preference of RDV-TP versus ATP incorporation into viral RNA, reducing the concentration of UTP required for incorporation opposite RDV on the complementary RNA strand, and reducing the steric clash between the polymerase and incorporated RDV-TP (24, 26, 30). RDV sensitivity can also be overcome by increasing concentrations of intracellular nucleotide pools (17). RDV resistance mutations identified *in vitro* in SARS-CoV-2 (nsp12-RdRp V792I and E802D) have been confirmed to emerge in patients with failure to respond to RDV demonstrating the value of *in vitro* testing of genetics and resistance pathways for prediction and surveillance (30, 64, 65).

Here, we show that a mutation in the MHV nsp13-HEL (A335V) contributes to RDV resistance. When the nsp13-HEL A335V substitution was combined with previously described nsp12-RdRp substitutions F476L and V553L, the resistance phenotype was additive. The A-to-V substitution at the homologous residue in SARS-CoV-2 nsp13-HEL (A336V) is a naturally occurring substitution present at low frequency in reported genomes in GISAID, so its impact on the SARS-CoV-2 protein was an important area of study (23). In biochemical experiments, the purified SARS-CoV-2 nsp13-HEL with the A336 substitution retained the ability to associate with the nsp7/nsp8_2_/nsp12 complex (Fig. 6C). In contrast, the SARS-CoV-2 nsp13-HEL A336V mutant protein had fourfold reduced unwinding rate of dsDNA and 4.5-fold reduced ATPase activity compared with WT nsp13-HEL.

### Critical determinants of helicase function in CoVs

CoV helicases have been shown to have multiple *in vitro* enzymatic activities including dNTPase and NTPase activities, 5′-3′ RNA and DNA unwinding that is enhanced by the presence of the nsp12-RdRp, and IFN antagonism (44, 47 - 50). Most studies to date have been of purified nsp13-HEL protein biochemistry and structural studies of the helicase alone or in association with nsps 7, 8, and nsp12-RdRp. The roles of the nsp13-HEL in CoV RNA synthesis and modification are just beginning to be addressed. The helicase catalytic residue K288 (K287 in MHV) has been reported to be essential for virus replication in the related Nidovirus PRRSV and in the CoVs HCoV-229E and Porcine Epidemic Diarrhea Virus (PEDV) (61, 62, 66). We have recently shown that in MHV, deletion of the nsp5-Mpro cleavage site between the nsp13-HEL and nsp14-exoribonuclease allows for recovery of mutant viruses that have impaired replication and fitness (67). There is also evidence that mutations in residues outside conserved helicase motifs in CoVs may alter helicase enzymatic function: the stalk domain has been shown to be essential for nsp13-HEL activity in SARS-CoV-2, and a mutation in the RecA1 domain, T216A, impaired helicase activity *in vitro* (52, 68).

### The MHV nsp13 A335V substitution in replication and tropism

In addition to helicase enzymatic activity, nsp13 has been described as a determinant of tropism. The MHV nsp13-HEL A335V substitution has been previously reported as a site of difference between related isolates of MHV with differing tissue tropism (51). MHV strain JHM.WU encodes the WT A335, while strain JHM.SD encodes V335. JHM.WU (A335) replicates to high titers in the murine liver and causes hepatitis, while JHM.SD (V335) infects neural tissue and does not replicate well or induce pathology in the liver (51). In this study, introduction of the A335V substitution into the cloned JHM.WU background attenuated replication kinetics and reduced viral titer both *in vivo* and *in vitro* in murine liver cells (51). This study showed that the A335V substitution can occur naturally in MHV and plays an additional role in tissue-specific viral replication *in vivo*. Combined, these multiple biological and biochemical functions of nsp13-HEL and its role in nucleoside analog resistance emphasize this protein’s importance in viral replication.

### Structural and biochemical evidence for nsp13-HEL interaction with the RTC

Structures of the SARS-CoV, MERS-CoV, and SARS-CoV-2 helicases, both alone and in association with nsps 7/8_2_/12, provide evidence for distinct protein-protein interfaces between the nsp13-HEL and nsp8 and nsp13-HEL and nsp12-RdRp (43, 52 - 54). Complexes of nsps 7/8_2_/12 can be bound to one or two protomers of nsp13-HEL, suggesting that the nsp13-HEL may have a dynamic association with the RTC and may function as a dimer during viral replication (43, 52 - 54). When one nsp13-HEL is bound to nsps 7/8_2_/12, its zinc-binding domain (ZBD) binds on the thumb side of the RdRp; when two protomers bind, one binds to the RdRp thumb, and one binds to the RdRp fingers domain (43). When bound, both copies of the nsp13-HEL sit on top of the nsps 7/8_2_/12 complex with each nsp13-ZBD interacting with one of the two N-terminal helical extensions of nsp8, and one of the nsp13-ZBD makes additional interactions with the nsp8 globular head (43). The CoV nsp13-HEL has also been shown to be a flexible protein with “open” and “closed” forms (54).

Modeling A336 onto solved structures of CoV helicases places the mutation in a surface-exposed region adjacent to the RNA binding channel (Fig. 1B). The A336V substitution replaces the methyl group with an isopropyl group, possibly causing steric hindrance in the RNA binding channel. Additionally, the more hydrophobic valine could cause the looped region of nsp13-HEL to alter its conformation to be less solvent-exposed. In structures of the SARS-CoV-2 nsp13-HEL in complex with nsps 7/8_2_/12-RdRp where two nsp13-HELs are bound, the A336 of one helicase is adjacent to the RNA binding channel, while the A336 of the other helicase is in a surface-exposed loop sitting above the nsp12-RdRp (43). The impacts of A336V on both unwinding and ATPase activities despite its distance from the known catalytic motifs governing these activities suggest that this residue may be a critical regulator of the helicase enzymatic activities and virus replication.

### Potential models for CoV helicase influence on RDV resistance

We have shown that the MHV nsp13-HEL A335V substitution confers RDV resistance and impairs helicase unwinding and ATPase activities. While these data do not directly demonstrate a mechanism for RDV resistance, they do support at least two possible strategies by which the A335V substitution could confer RDV resistance. First, in CoVs, it has been demonstrated that RDV incorporation can be competitively overcome by increasing concentrations of NTP pools (17). In the alphavirus Chikungunya virus, the viral helicase/protease has been shown to regulate replication fidelity by altering nucleotide pools (69). A mutation in the Chikungunya virus helicase/protease metal-binding region resulted in impaired helicase and NTPase activity *in vitro* but increased polymerase activity and reduced viral mutation frequency (69). This work highlighted that nucleoside analog incorporation by Chikungunya virus was dependent on conditions of nucleotide depletion and that increasing exogenous nucleoside pools was sufficient to restore viral growth in the presence of nucleoside analog antivirals (69). By analogy, if the MHV A335V substitution impairs ATPase activity resulting in increased local ATP concentrations in double-membrane-bound CoV replication factories, this could favor ATP incorporation over RDV, resulting in a relative resistance phenotype. Alteration of helicase processivity is another potential mechanism by which the A335V substitution could confer partial RDV resistance. In the structurally related human SF1 helicase UPF-1, a protein involved in multiple pathways of nucleic acid metabolism, mutations outside canonical catalytic motifs have been shown to alter nucleic acid binding and helicase processivity and subsequent nucleic acid metabolism (70). In CoVs, nsp12-RdRp processivity and the processivity of the entire replication transcription complex (RTC) is likely critical for efficient replication of the large CoV genome (41). CoV replication is further defined by the proofreading ability of the nsp14-exoribonuclease (nsp14-ExoN), which is required for high-fidelity replication and regulates CoV sensitivity to mutagens (71). If the demonstrated impaired unwinding of the mutant helicase also impairs translocation of the replicase along template RNA, the slowed RTC processivity could allow additional opportunity for nucleotide selectivity by the nsp12-RdRp or for the proofreading nsp14-ExoN to excise RDV from product RNA.

The results of this study indicate that the nsp13-HEL is a key determinant of helicase function and RNA synthesis. We highlight a residue outside canonical helicase enzymatic motifs that impairs unwinding and ATPase activity and demonstrates a novel role for the nsp13-HEL in RDV resistance. We note that we performed all genetic studies in MHV but did not attempt the homologous substitution in SARS-CoV-2 based on our interpretation of the *NIH Guidelines for Research Involving Recombinant or Synthetic Nucleic Acid Molecules* (Section III-A-1-a; April 2019) that limits or prevents reverse genetic introduction of putative resistance mutations against approved (or EUA) antivirals such as RDV into a pandemic pathogen such as SARS-CoV-2 (72). To date the SARS-CoV-2 nsp13-HEL A336V mutation has not been described in RDV-treated patients or in our or other studies on *in vitro* passage of SARS-CoV-2 with RDV. Despite this external constraint, our combination *in vitro* studies of the SARS-CoV-2 helicase biochemistry and MHV viral genetics support that the nsp13-HEL would be a likely high-profile target for antiviral development, either alone or in combination with nucleoside analogs or protease inhibitors. It also is critical to continue surveillance for mutations in the SARS-CoV-2 nsp13-HEL from RDV-treated patients and to define the role of A336 and other helicase mutations in CoV replication and pathogenesis.

## MATERIALS AND METHODS

### Cell culture

Murine astrocytoma delayed brain tumor (DBT-9) cells and baby hamster kidney 21 cells were maintained at 37°C in Dulbecco’s modified Eagle medium (DMEM) (Gibco) supplemented with 10% fetal bovine serum (FBS) (Invitrogen), 1% penicillin and streptomycin (Gibco), and 0.1% amphotericin B (Corning).

### Viruses and amino acid conservation

All work with MHV was performed using the recombinant WT strain MHV-A59 (GenBank accession number AY910861, https://www.ncbi.nlm.nih.gov/nuccore/AY910861). To determine conservation across multiple CoVs, multiple sequence alignments were generated using MacVector.

### Compounds

Remdesivir MedChem Express (RDV) was prepared as a 20 mM stock solution in DMSO. EIDD-1931 was synthesized at the Emory Institute for Drug Development and prepared as a 20 mM stock solution in DMSO.

### Cloning, recovery, and verification of mutant viruses

QuikChange mutagenesis was performed according to the manufacturer’s protocol to generate mutations in MHV individual genome cDNA fragment plasmids using the previously described infectious clone reverse-genetics system (73). Mutants were recovered in BHK-R cells following electroporation of *in vitro*-transcribed genomic RNA. All fragments containing mutations as well as virus stocks were sequenced to ensure mutations were present before use in further studies (GenHunter).

### Model building of MHV nsp13

A model of the MHV nsp13 was generated by ColabFold using the MHV_A59 sequence (YP_009724389.1) (57, 58). The results were then visualized and aligned to the SARS-CoV-2 nsp13.1 from the solved structure 6XEZ in PyMOL (Schrödinger).

### Alignment of betacoronavirus nsp13 by BLAST

The following sequences were used for reference to compare amino acid identity by NCBI Basic Local Alignment Search Tool (BLAST): MHV_A59 (YP_009724389.1), HCoV HKU1 (YP_173236.1), HCoV OC-43 (YP_009555238.1), MERS-CoV (YP_009047224.1), SARS-CoV (YP_009725308.1), and SARS-CoV-2 (YP_009724389.1).

### Illumina RNA-sequencing of viral RNA

Total RNA was extracted from MHV WT, P1, P5, P10, P15, P20, and P23 monolayers using TRIzol according to the manufacturer’s instructions. For RNA-Seq, total RNA underwent poly(A) selection followed by NovaSeq PE150 sequencing (Illumina) at 15 million reads per sample at the Vanderbilt University Medical Center (VUMC) core facility, Vanderbilt Technologies for Advanced Genomics (VANTAGE). Reads were aligned to the reference genome (AY910861), and mutations were identified, quantified, and annotated using the in-house pipeline, CoVariant. Amino acid locations were confirmed through sequence alignment using MacVector and CLC Workbench (QIAGEN).

### Plaque purification of plaque isolates

Supernatant collected from passaging MHV in the presence of GS-441524 was serially diluted and used to infect sub-confluent monolayers of DBT-9 cells. 24 hpi, a plaque, was selected with a sterile pipet tip and placed in 1 mL warm DMEM. 500 µL of this sample was then used to infect a T25 flask of DBT-9 cells to generate a P1 of the plaque pick. Plaque isolates were purified in triplicate in this manner and then sequenced.

### Virus replication assays

Sub-confluent monolayers of DBT cells were infected at an MOI of 0.01 PFU/cell for 1 h (MHV). Inocula were removed, and the cells were washed with PBS before addition of prewarmed medium. Supernatants were harvested at the indicated times post-infection and titers were determined by plaque assay.

### Plaque assays

Plaque assays were performed in sub-confluent DBT-9 cells seeded in 6-well plates. Serial dilutions were plated in duplicate and overlaid with 1% agar in DMEM. Titers were scored at 24 hpi.

### Quantification of viral genomic RNA

Subconfluent DBT cells were infected with WT-MHV at an MOI of 0.01 PFU/cell. The inoculum was removed after 1 h of incubation at 37°C, and a medium containing the indicated concentration of nucleoside analogue was added. Total RNA from cells and supernatant RNA were harvested using TRIzol reagent (Invitrogen) after 20 h. Both total RNA and supernatant RNA were extracted by phase separation. Total RNA was purified by ethanol precipitation, and supernatant RNA was purified using a PureLink RNA minikit (Invitrogen) according to the manufacturer’s protocol. Total RNA was reverse transcribed using SuperScript III (Invitrogen) to generate cDNA, which was quantified by quantitative PCR (qPCR) as previously described (58). Data are presented as 2^−ΔΔCT^, where ΔΔCT denotes the change in the threshold cycle for the viral target (nsp10) normalized to the control [glyceraldehyde-3-phosphate dehydrogenase (GAPDH)] before and after drug treatment. The supernatant RNA was quantified using one-step quantitative reverse transcriptase PCR (qRT-PCR). The data are presented as the fold change in genome RNA copies normalized to vehicle control.

### Competitive fitness assay

The MHV competitive fitness assay was previously described in detail (56). Briefly, sub-confluent DBT-9 cells were co-infected with the indicated virus (WT, p23, nsp13-A335V, or nsp12-RdRp F476L + V553L + nsp13-HEL A335V) and a barcoded WT-MHV reference virus with seven silent mutations in nsp2 (1301-CAGCAGT-1307) at a total MOI of 0.1 PFU/cell (0.05 MOI for each virus) in three independent lineages. The resulting virus was passaged four additional times, each at a constant MOI of 0.1 PFU/cell. Viral RNA from each passage supernatant was extracted in TRIzol at purified with a KingFisher II (ThermoFisher Scientific) according to the manufacturer’s protocol. RNA corresponding to the barcoded WT reference and test viruses was determined by one-step RT-qPCR using SYBR green. BC WT reference RNA was detected with forward primer (5′-CTATGCTGTATACGGACAGCAGT) and reverse primer (5′-GGTGTCACCACAACAATCCAC) using a Power SYBR green RNA-to-Ct 1-step kit (Applied Biosystems) on a StepOnePlus real-time PCR system (Applied Biosystems). The log-transformed cycle threshold (C_T_) ratio of test versus reference was plotted over passage, and relative fitness was determined by comparing the slopes of the linear regression (58, 67, 74).

### Nucleoside analogue sensitivity studies

Sub-confluent monolayers of DBT cells were infected with MHV at an MOI of 0.01 PFU per cell for 1 h at 37°C. The inoculum was removed and replaced with a medium containing the indicated compound concentration. Cell supernatants were harvested 24 h post-infection. Titers were determined by plaque assay as described previously.

### Expression and purification of WT/A336V nsp13-HEL

SARS-CoV-2 nsp13 WT and A336V utilized in biochemical assays were expressed and purified as previously reported (42, 53). The pET28a plasmid containing SARS-CoV-2 His_6_-PreScission-nsp13 (Addgene plasmid #159390) was used as the WT backbone. Site-directed mutagenesis using the Q5 SDM kit was performed utilizing an altered forward primer (5′-CACGCATCATCCCAGTTAGGGCCAGAGTCGA ATGCTTCGACAAGTTCAAGG-3′) to obtain the mutant plasmid. Plasmids were transformed into *E. coli* Rosetta (DE3) cells (Novagen) and plated on LB-agar containing 50 and 25 µg/mL of kanamycin and chloramphenicol, respectively. Cells were grown in LB media to OD_600_ = 0.6 at 37°C at 200 rpm, induced with 0.2 mM IPTG, then incubated for 18 h at 16°C. Cells were collected via centrifugation, resuspended (50 mM HEPES pH 8.0, 500 mM NaCl, 20 mM Imidazole, 5 mM MgCl_2_, 5% glycerol (v/v), 5 mM BME, 1× Protease Inhibitor Cocktail (Roche), 1 mM PMSF), and lysed through a French Press (Avestin). The cleared lysate was loaded onto HisTrap HP column (Cytiva), washed, and eluted (50 mM HEPES pH 8.0, 500 mM NaCl, 250 mM Imidazole, 5 mM MgCl_2_, 5% glycerol, and 5 mM BME). Eluate was dialyzed overnight with PreScission Protease to cleave the His_6_-tag. Cleaved proteins were passed through another HisTrap HP column, and the resulting flow-through was injected onto a Superdex 200 Hiload 16/600 (Cytiva) for size-exclusion chromatography (50 mM HEPES pH 8.0, 500 mM NaCl, 5 mM MgCl_2_, 5% glycerol, and 1 mM DTT). Glycerol was added to purified nsp13 (to reach 20% final), aliquoted, flash-frozen with liquid N_2_, and stored at −80°C until use.

### Native EMSA

nsp12 was expressed and purified as previously reported (43). nsp12 was incubated with 2.5× molar excess of nsp7/8 at RT for 15 min to assemble a tripartite complex of nsps 7/8_2_/12 before being buffer exchanged into assay buffer (120 mM K-acetate pH, 20 mM HEPES pH 8.0, 10 mM MgCl_2_, 2 mM DTT) via a Zeba Spin Desalting Column (7 kDa MWCO; ThermoFisher). dsRNA scaffold was created by combining the primer (5′-CGCGUAGCAUGCUACGUCAUUCUCCUAAGAAGCUAC-3′) and template (5′-CUAUCCCCAUGUGAUUUUAAUAGCUUCUUAGGAGAAUGACGUA GCAUGCUACGCG-3′) strands in 10× annealing buffer, heating to 95°C for 2 min, and cooling to 10°C over 30 min. All oligos were purchased from Integrated DNA Technologies. The complex was incubated in an equimolar ratio with 1.5 µM annealed dsRNA scaffold (Horizon Discovery) at 37°C for 15 min. nsp13s and premixed ADP-AlF_3_ (Sigma-Aldrich) were added to a final concentration of 3 µM and 2 mM, respectively, and incubated for an additional 5 min at 30°C. Reactions were analyzed by native gel electrophoresis on a 4.5% polyacrylamide native gel (37.5:1 acrylamide:bis-acrylamide) in 1× TBE (89 mM Tris, 89 mM boric acid, and 1 mM EDTA) at 4°C. The gel was stained with Gel-Red (Biotium) to visualize nucleic acids.

### Helicase unwinding assay

The preparation of nucleic acid substrates and performing bulk helicase assays reflect the protocol described previously (49). All oligos were purchased from Integrated DNA Technologies. The DNA overhang template strand with the fluorophore (5′–TTTTTTTTTTCTGATGTTAGCAGCTTCGT/36-TAMSp/–3′) and the quencher containing primer (5′–/5lAbRQ/ACGAAGCTGCTAACATCAG–3′) were mixed in a 1:1.2 ratio, heated to 95°C for 5 min and cooled to 25°C at a rate of 1°C/min. RNA overhang template (5′-UUUUUUUUUUCUGAUGUUAGCAGCUUCGU/36 -TAMSp/–3′) and primer (5′–/5lAbRQ/ACGAAGCUGCUAACAUCAG –3′) were mixed in a 1:1.2 ratio, heated to 75°C for 5 min, and cooled to 25°C at a rate of 1°C/min.

For unwinding kinetics, reactions and their components were prepared in helicase buffer (20 mM HEPES pH 7.5, 40 mM KCl, 5 mM MgCl_2_, 0.5 mM EDTA, 1 mM DTT, 0.5 mM glutathione, 0.01% Triton X-100, and 0.1 mg/mL bovine serum albumin). For each well, 10 nM of nsp 13 (WT or A336V) was incubated for 10 min with dsDNA (0–4 µM) or dsRNA (0–6 µM) and 8 µM or 12 µM of excess capture DNA (5′– CTGATGTTAGCAGC TTCGT–3′), respectively, in a flat-black, 384-well microplate (Grenier Bio-One). Plates were centrifuged at 1,000 rpm for 1 min before being loaded on a Synergy Neo2 plate reader (Biotek), which dispensed ATP (2 mM final) to a final volume of 24 µL and initiate the reactions. Substrate unwinding was measured continuously by recoding changes in fluorescence (excitation: 540 ± 20 nm and emission: 603 ± 20 nm) in 5 s intervals over 10 min. Background signal from wells without ATP but with corresponding amount of unwinding substrate was subtracted from experimental wells. Standard curves for each substrate were generated by TAMRA-containing template strands only and utilized to convert relative fluorescence units (RFUs) to units of molarity. The first 150 s and 500 s for DNA and RNA, respectively, were graphed to a hyperbolic fit on Prism (GraphPad Software) where V_max_ and K_m_ values were used to derive points for Michaelis-Menten curve fit to obtain enzymatic parameters.

### NADH-coupled ATPase assay

Reaction components were prepared in assay buffer (20 mM HEPES, pH 7.5, 40 mM KCl, 5 mM MgCl_2_, 2.5 mM glutathione, 0.5 mM EDTA, 0.01% Triton X-100, and 0.1 mg/mL bovine serum albumin), and reactions were carried out in a flat-black, 384-well microplate (Greiner Bio-One). Purified SARS-CoV-2 nsp13-HEL was first buffer-exchanged into helicase buffer (20 mM HEPES pH 7.5, 40 mM KCl, 5 mM MgCl_2_, 0.5 mM EDTA, 1 mM DTT, 0.5 mM glutathione, 0.01% Triton X-100, and 0.1 mg/mL bovine serum albumin) using a Zeba Spin Desalting column (7K MWCO; Thermofisher) and then diluted in assay buffer. For each well, 10 mM of nsp13-HEL (WT or A336V) was incubated with a NADH oxidizing master mix containing phosphoenolpyruvate (PEP) (1 mM), pyruvate kinase (30 U/mL), and NADH (0.175 mM), and then subsequently spun down using a plate spinner (Thermoscientific) for 30 s at 1,000 rpm. ATP at the indicated concentrations, ranging from 1 mM to 0.97 µM, was added to each well, resulting in a final reaction volume of 24 µL. After a 30 s spin, the plate was placed in a Synergy Neo Plate reader (BioTek). As ATP hydrolysis is coupled to NADH oxidation, ATP hydrolysis could be monitored by measuring the decrease in fluorescence (excitation: 340 ± 20 nm and emission: 445 ± 20 nm) over 40 min. The fluorescence (RFU) for each well was plotted against time (s), and the linear portion of each curve was plotted using Prism 9 (GraphPad Software). The overall rate of fluorescence decrease for each reaction (slope) was calculated using the linear regression function. The rate of decreased fluorescence due to background NADH oxidation was determined from the control well without ATP and was subtracted from the slope of each experimental condition. The resulting inverse slope of change in fluorescence was converted to the rate of ATP hydrolysis using a conversion factor determined from an NADH standard curve and was plotted against ATP concentration in Prism (GraphPad Software) to a hyperbolic fit to determine the Michaelis-Menten curve and obtain enzymatic parameters such as V_max_ and K_m_.

### Statistics

Statistical tests were performed using GraphPad (La Jolla, CA) Prism 9 software as described in the respective figure legends.

## Data Availability

FASTQ files for the RNA-seq variant analysis have been deposited in the National Center for Biotechnology Information Sequence Read Archive (NCBISRA) under the accession number PRJNA922123. All code used in this study can be accessed at https://github.com/DenisonLabVU.

## SUPPLEMENTAL MATERIAL

Fig S1 mbio.01060-23-s0001.pdf Progress curves for ATPase assay.

Table S1 mbio.01060-23-s0002.pdf SARS-CoV-2 variants at A336.

Table S2 mbio.01060-23-s0003.pdf Kinetics for unwinding assay.

Table S3 mbio.01060-23-s0004.pdf Kinetics for ATPase assay.

ASM does not own the copyrights to Supplemental Material that may be linked to, or accessed through, an article. The authors have granted ASM a non-exclusive, world-wide license to publish the Supplemental Material files. Please contact the corresponding author directly for reuse.
