# Supplementary material for: A mutation in the coronavirus nsp13-helicase impairs enzymatic activity and confers partial remdesivir resistance
Source: mBio. 2023 Jun 20;14(4):e01060-23. doi: 10.1128/mbio.01060-23 (PMC10470589; doi:10.1128/mbio.01060-23)
Supplement: Fig S1 — Progress curves for ATPase assay. [file mbio.01060-23-s0001.pdf]

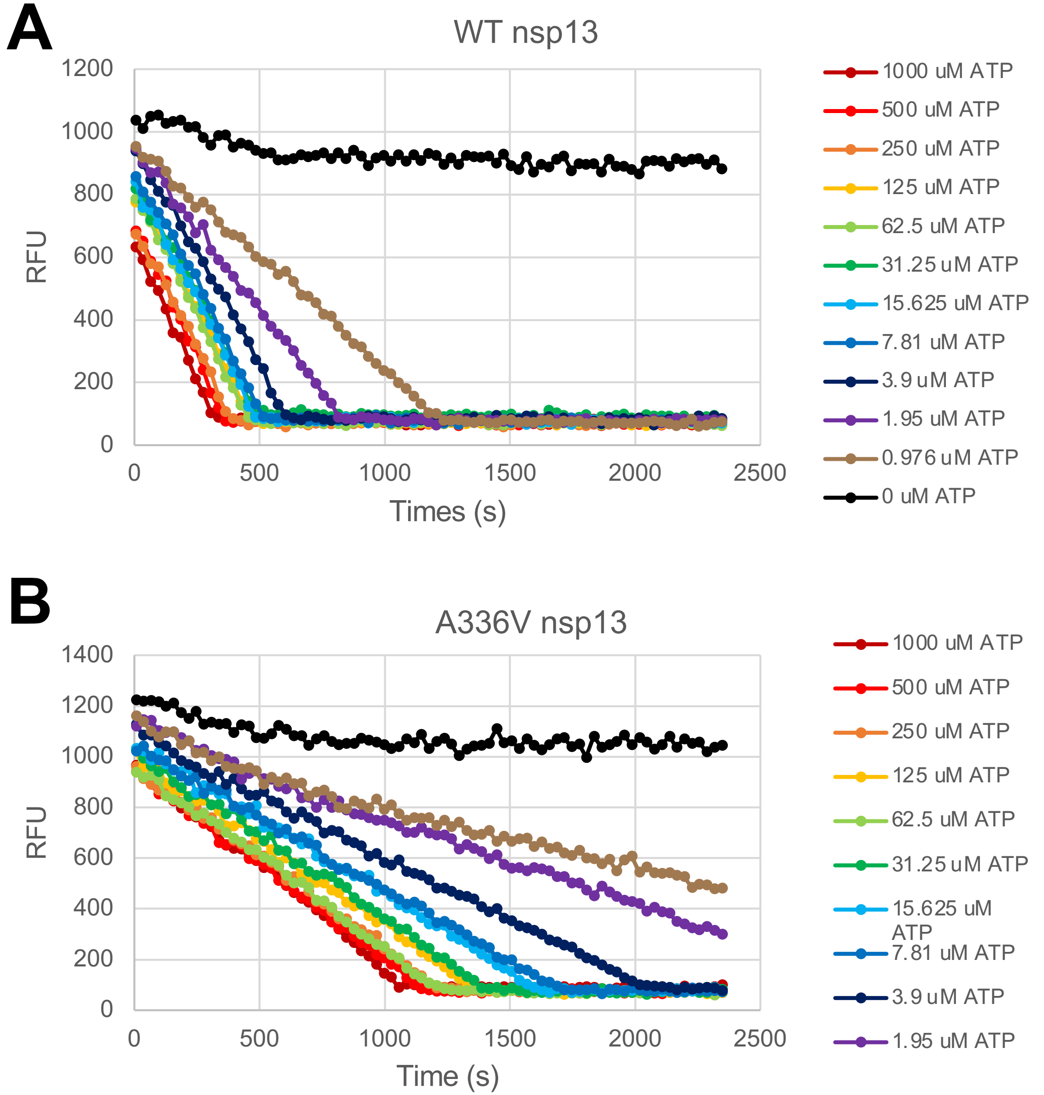


**Supplementary Figure 1. SARS-CoV-2 helicase with the A336V substitution has reduced ATPase activity. (A and B)** Representative progress curves of ATPase activity for WT (A) and mutant nsp13-HEL (B). Fluorescence (RFU) (excitation: 340 ± 20 nm and emission: 445 ± 20 nm) was measured over time (s). Linear portion of each curve was used to determine the rate of NADH oxidation and, therefore, ATP hydrolysis.
