# Supplementary material for: A mutation in the coronavirus nsp13-helicase impairs enzymatic activity and confers partial remdesivir resistance
Source: mBio. 2023 Jun 20;14(4):e01060-23. doi: 10.1128/mbio.01060-23 (PMC10470589; doi:10.1128/mbio.01060-23)
Supplement: Table S1 — SARS-CoV-2 variants at A336. [file mbio.01060-23-s0002.pdf]

<u>Substitution</u>	**Total Seq. Reported**	<u>Substitution</u>	**Total Seq. Reported**	<u>Substitution</u>	**Total Seq. Reported**
A336_	9,373	A336L	27	A336E	2
A336S	5,919	A336R	4	A336H	1
A336T	2,388	A336Y	4	A336K	1
**A336V**	**888**	A336I	3	A336G	-
A336C	81	A336F	2	A336N	-
A336D	49	A336M	2	A336P	-
A336del	45	A336Q	2	A336W	-

Supplementary Table 1: nsp13-HEL A336 is a site of variation in SARS-CoV-2 sequences reported in GISAID. As of May 1, 2023, GISAID reported 15,493,847 SARS-CoV-2 genomes. Of these, 9,373 isolates were reported with a coding change at residue A336, including over 800 A336V substitutions. The first reported isolate with an A336 substitution (A336T) was collected in March 2020 in New York, USA, and the first A336V substitution was reported in April 2020 in Castilla-La Mancha, Spain. These reported isolates are not necessarily indicative of viable virus, however, and are based on inherently biased sampling and reporting data.
