# Supplementary material for: A mutation in the coronavirus nsp13-helicase impairs enzymatic activity and confers partial remdesivir resistance
Source: mBio. 2023 Jun 20;14(4):e01060-23. doi: 10.1128/mbio.01060-23 (PMC10470589; doi:10.1128/mbio.01060-23)
Supplement: Table S2 — Kinetics for unwinding assay. [file mbio.01060-23-s0003.pdf]

	WT + RNA	A336V + RNA	WT + DNA	A336V + DNA
**Best Fit Values**				
V_{max}	26.99	18.21	35.10	21.45
Km	6216	6682	240.3	541.5
**95% CI**				
V_{max}	24.04 to 30.86	9.181 to +infinity	30.66 to 40.17	17.45 to 27.02
K_m	5109 to 7703	1843 to +infinity	135.5 to 400.3	265.2 to 1083
**Goodness of Fit**				
Degree of Freedom	13	13	22	21
R squared	0.9948	0.7750	0.6980	0.7207
Sum of Squares	1.459	36.39	591.8	235.1
Sy.x	0.3350	1.673	5.186	3.346
**Constraints**				
K_m	Km > 0	Km > 0	Km > 0	Km > 0
**Number of Points**				
# of X values	44	44	24	24
# of Y values analyzed	15	15	24	23

Supplementary Table 2: Statistical parameters for nsp13-HEL WT and A336V Unwinding activity with dsRNA versus dsDNA substrates. Table summarizing the statistical parameters of the Michaelis-Menten Curves (Fig. 7) depicting nsp13-HEL WT and A336V unwinding activity as a function of dsRNA or dsDNA substrate concentration.
