# Supplementary material for: A mutation in the coronavirus nsp13-helicase impairs enzymatic activity and confers partial remdesivir resistance
Source: mBio. 2023 Jun 20;14(4):e01060-23. doi: 10.1128/mbio.01060-23 (PMC10470589; doi:10.1128/mbio.01060-23)
Supplement: Table S3 — Kinetics for ATPase assay. [file mbio.01060-23-s0004.pdf]

	WT nsp13	A336V nsp13
**Best Fit Values**		
V_{max}	0.3421	0.1066
Km	1.881	2.616
**95% CI**		
V_{max}	0.3247 to 0.3598	0.09488 to 0.1190
K_m	1.357 to 2.557	1.244 to 5.116
**Goodness of Fit**		
Degree of Freedom	21	22
R squared	0.9414	0.7761
Sum of Squares	0.01666	0.007789
Sy.x	0.02816	0.01882
**Constraints**		
K_m	Km > 0	Km > 0
**Number of Points**		
# of X values	36	36
# of Y values analyzed	23	24

Supplementary Table 3: Statistical parameters for nsp13-HEL WT and A336V ATPase activity. Table summarizing the statistical parameters of the Michaelis-Menten Curves (Fig. 8) depicting nsp13-HEL WT and A336V ATPase activity as a function of ATP concentration.
